# The Mixture of Salvianolic Acids from *Salvia miltiorrhiza* and Total Flavonoids from *Anemarrhena asphodeloides* Attenuate Sulfur Mustard-Induced Injury

**DOI:** 10.3390/ijms161024555

**Published:** 2015-10-15

**Authors:** Jianzhong Li, Linlin Chen, Hongyuan Wu, Yiming Lu, Zhenlin Hu, Bin Lu, Liming Zhang, Yifeng Chai, Junping Zhang

**Affiliations:** 1 School of Pharmacy, Second Military Medical University, Shanghai 200433, China; E-Mails: llchen1015@163.com (L.C.); xingfude.women@163.com (H.W.); bluesluyi@sina.com (Y.L.); zhenlinhu@hotmail.com (Z.H.); binlu@smmu.edu.cn (B.L.); yfchai@smmu.edu.cn (Y.C.); 2 Department of Chemical Defence Medicine, Faculty of Navy Medicine, Second Military Medical University, Shanghai 200433, China; E-Mail: 13818628872@139.com

**Keywords:** sulfur mustard, glutathione, gene expression, signaling pathway

## Abstract

Sulfur mustard (SM) is a vesicating chemical warfare agent used in numerous military conflicts and remains a potential chemical threat to the present day. Exposure to SM causes the depletion of cellular antioxidant thiols, mainly glutathione (GSH), which may lead to a series of SM-associated toxic responses. MSTF is the mixture of salvianolic acids (SA) of S*alvia miltiorrhiza* and total flavonoids (TFA) of *Anemarrhena asphodeloides*. SA is the main water-soluble phenolic compound in S*alvia miltiorrhiza.* TFA mainly includes mangiferin, isomangiferin and neomangiferin. SA and TFA possess diverse activities, including antioxidant and anti-inflammation activities. In this study, we mainly investigated the therapeutic effects of MSTF on SM toxicity in Sprague Dawley rats. Treatment with MSTF 1 h after subcutaneous injection with 3.5 mg/kg (equivalent to 0.7 LD_50_) SM significantly increased the survival levels of rats and attenuated the SM-induced morphological changes in the testis, small intestine and liver tissues. Treatment with MSTF at doses of 60 and 120 mg/kg caused a significant (*p* < 0.05) reversal in SM-induced GSH depletion. Gene expression profiles revealed that treatment with MSTF had a dramatic effect on gene expression changes caused by SM. Treatment with MSTF prevented SM-induced differential expression of 93.8% (973 genes) of 1037 genes. Pathway enrichment analysis indicated that these genes were mainly involved in a total of 36 pathways, such as the MAPK signaling pathway, pathways in cancer, antigen processing and presentation. These data suggest that MSTF attenuates SM-induced injury by increasing GSH and targeting multiple pathways, including the MAPK signaling pathway, as well as antigen processing and presentation. These results suggest that MSTF has the potential to be used as a potential therapeutic agent against SM injuries.

## 1. Introduction

Sulfur mustard (SM) or bis(2-chloroethyl) sulfide is a powerful bifunctional alkylating and vesicating chemical warfare agent used in numerous military conflicts during the 20th century [1,2]. SM is known as a blistering agent, first introduced as a weapon during World War I. Exposure to SM causes the depletion of cellular antioxidant thiols, mainly glutathione (GSH), which may lead to accumulation of reactive oxygen species (ROS) and reactive nitrogen species within cells, inactivation of sulfhydryl-containing enzymes, loss of calcium homeostasis, lipid peroxidation, cellular membrane breakdown and, ultimately, cell death [3,4,5,6,7]. These events lead to oxidative stress and macromolecular damage, including DNA, RNA and protein damage, which trigger intricate signaling pathways and modulate gene expression, causing a series of SM-associated toxic responses [5,6,8,9]. In addition, SM or CEES (2-chloroethyl ethyl sulfide, SM analog) caused oxidative stress results in the 8-oxo-2-deoxyguanosine DNA adduct, as well as lipid and protein oxidation, which can cause inflammation and other toxic responses in skin [10,11].

SM is hydrophobic in nature. Therefore, it easily penetrates and accumulates in the lipid component of exposed tissues. In case of contact with skin, SM not only accumulates in the skin, but also distributes to other tissues, such as brain, kidney, muscle, spleen, liver and bone marrow, through the systemic circulation [12]. SM-induced injuries can take several months to heal and require long-term medical treatment. Exposure to SM triggers an array of complex signal transduction pathways, suggesting the need for pleiotropic agents or combination therapies to treat SM-induced injuries. This is consistent with treating diseases with multi-components in traditional Chinese medicine. The exogenous addition of antioxidants, such as GSH, *N*-acetyl cysteine (NAC), vitamin E, superoxide dismutase (SOD), catalase, sulforaphane and quercetin, was reported to attenuate lung and skin injury by SM/CEES [5,11,13,14,15]; however, most of them exhibit a stronger protective effect than therapeutic potential [5,7,16]. Due to the potential for use of SM as a chemical terrorism agent, there is renewed interest in developing effective chemopreventive and therapeutic agents.

In this study, we aimed to investigate the therapeutic effects of the mixture of SA and TFA (MSTF) on SM toxicity and to elucidate the potential molecular mechanisms of MSTF-mediated therapeutic efficacy in Sprague Dawley rats.

## 2. Results

### 2.1. Effect of MSTF on Seven-Day Survival

**Figure 1:**
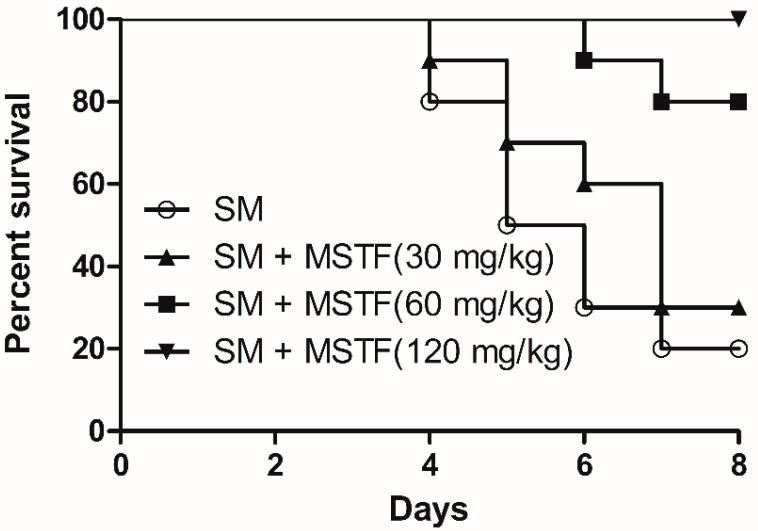
Effect of MSTF (the mixture of SA and TFA) on survival time following sulfur mustard (SM) subcutaneous injection in rats. Survival of rats (*n* = 10 per group) treated with MSTF (30, 60 and 120 mg/kg) once each day for seven consecutive days 1 h after 3.5 mg/kg SM treatment. The survival curves were significantly different as predicted by the log rank test (*p* = 0.0008).

The effect of MSTF on survival time following SM subcutaneous injection is presented in Figure 1. Survival time was calculated on the basis of a seven-day observation period. In general, the rats began to die on the fourth day after SM subcutaneous injection. Treatment with MSTF at doses of 30, 60 and 120 mg/kg for seven consecutive days after SM subcutaneous injection significantly increased the survival levels of rats compared to the SM-alone group (Figure 1). They were 30% at 30 mg/kg, 80% at 60 mg/kg and 100% at 120 mg/kg for the MSTF treated groups, as compared to 20% in the corresponding SM-only group. The log rank test indicated that the survival curves were significantly different (*p* = 0.0008).

### 2.2. Effect of MSTF on MDA, SOD and GSH

The effect of MSTF on the level of malondialdehyde (MDA), SOD and GSH in the blood seven days after exposure to SM is shown in Table 1. The level of GSH was significantly decreased in the SM group compared to the control. Treatment with MSTF at doses of 60 and 120 mg/kg significantly increased GSH as compared to the SM-only group. However, the levels of SOD and MDA were not significantly altered following subcutaneous injection with SM.

**Table 1:** Effect of MSTF on the antioxidant status in rat blood (*n =* 10 rats/group). The measurement of MDA, SOD and GSH was carried out 7 days after the application of SM (mean ± SD).

Groups	MDA (μmol/mL)	SOD (U/mL)	GSH (µmol/mL)
Control	1.81 ± 0.82	6.13 ± 1.89	7.13 ± 1.26
SM	2.25 ± 0.57	6.22 ± 1.78	4.15 ± 0.77 ^a^
SM + MSTF (30 mg/kg)	2.34 ± 0.61	5.33 ± 1.87	3.37 ± 1.52 ^a^
SM + MSTF (60 mg/kg)	2.32 ± 0.54	5.14 ± 1.21	5.23 ± 1.67 ^b^
SM + MSTF (120 mg/kg)	4.85 ± 0.91 ^a,b^	4.1 ± 1.71	6.86 ± 1.41 ^b^

^a^ *p* < 0.05 as compared to the control group; ^b^ *p* < 0.05 as compared to the SM-only group.

### 2.3. Histologic Observations of the Testis, Small Intestine and Liver Tissues

**Figure 2:**
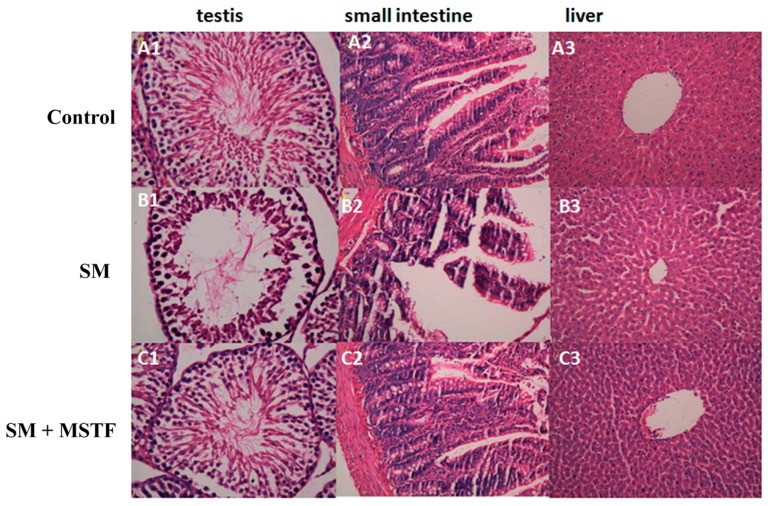
Representative photographs showing the histopathologic changes in testis, small intestine and liver tissues on the third day post-exposure (*n =* 5 rats/group). (**A1**–**A3**) Control (normal) group; (**B1**–**B3**) SM-treated group: the decrease of sperm cells, the villus epithelial shedding of small intestinal cells, the defects of the gut mucosal barrier, as well as the swelling of liver cells; (**C1**–**C3**) SM + MSTF group treated with MSTF (120 mg/kg) 1 h after SM treatment. The damages were ameliorated by MSTF treatment (H&E, 40×).

No obvious anomalies were seen in the testis, small intestine and liver tissues of normal control group rats (Figure 2). Significant pathological changes occurred at three days after SM exposure. Damage in testis, liver and small intestine, such as the decrease of sperm cells, the villus epithelial shedding of small intestinal cells, the defects of the gut mucosal barrier, as well as the mild swelling of liver cells, were observed, respectively (Figure 2B1–3). The damages were ameliorated by MSTF treatment at a dose of 120 mg/kg in the testis, liver and small intestine (Figure 2C1–3). These results illustrated that treatment of rats with MSTF after exposure to SM significantly attenuated the SM-induced morphological changes in the testis, small intestine and liver tissues, confirming the therapeutic effects of MSTF on SM-induced tissue injury.

### 2.4. MSTF Prevents SM-Induced Differential Expression of Many Genes

**Figure 3:**
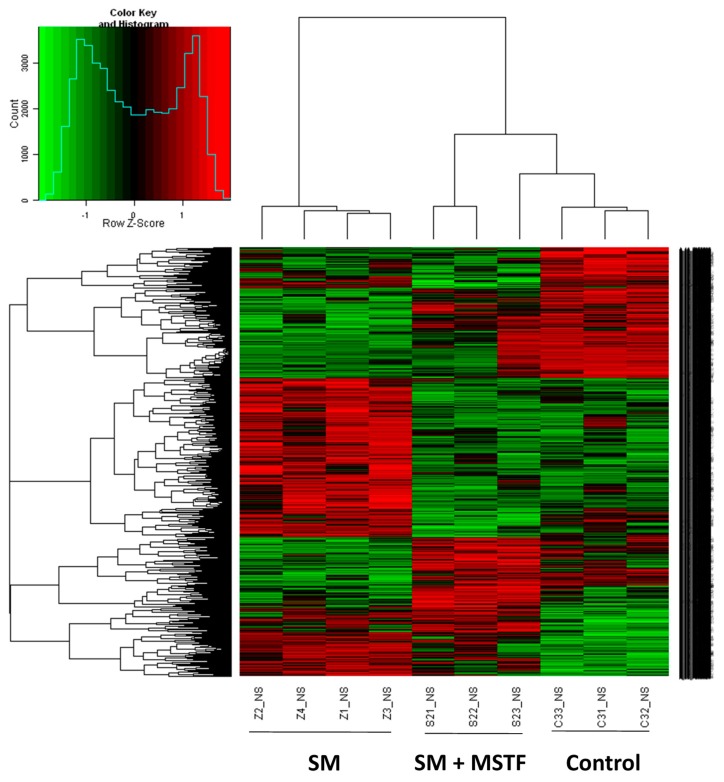
Hierarchical cluster analysis of individual rats according to the profile of gene expression examined. The whole genome microarray was conducted on rat liver tissues 24 h after the application of SM. Then, 4869 transcripts (probe sets) were organized by hierarchical clustering based on overall similarity in expression patterns. Red represents relative expression greater than the median expression level across all samples, and green represents an expression level lower than the median. Black indicates intermediate expression. Rats from the same treatment group are the closest. The control group is close to the SM + MSTF group treated with MSTF (120 mg/kg) 1 h after SM treatment and clearly separate from the SM-only-treated group.

The liver is considered to be a major target organ of SM-induced toxicity [17]. To further investigate the potential molecular basis of the therapeutic effects of MSTF on SM-induced damage, gene expression analysis was conducted on rat liver tissues 24 h after the application of SM using microarrays. A heatmap is generated (Figure 3) representing 4869 transcripts (probe sets) comparing significantly differentially regulated genes (*p* < 0.05) for each treatment condition (SM, SM + MSTF) *versus* untreated controls by using one-way analysis of variance (one-way ANOVA) (Table S1 in the Supplementary Information). Cluster analysis of the microarray results revealed that three or four rats from the same group were the closest, indicating that all of the experimental data in this study are reliable.

A combined algorithm with a simple Student’s *t*-test and fold change was used to find differentially-expressed genes. By a threshold of *p* < 0.05 and absolute fold change (FC) ≥ 1.5, 1037 differentially-expressed genes (transcripts) were selected by comparison between SM and wild-type (control) samples (Table S2 in the Supplementary Information). Among the genes originally displaying FC ≥ 1.5, 590 were downregulated and 447 upregulated. In the treatment with MTSF groups, only 64 out of 1037 genes were still differentially expressed in response to SM. These results indicate that treatment with MSTF prevented SM-induced differential expression of 93.8% (973 genes) of the genes (Table S3 in the Supplementary Information). We focused on these genes for which SM-induced alteration of expression was abolished or attenuated by MSTF treatment, as these genes might be regulated by MSTF and involved in the protective effects on SM.

**Figure 4:**
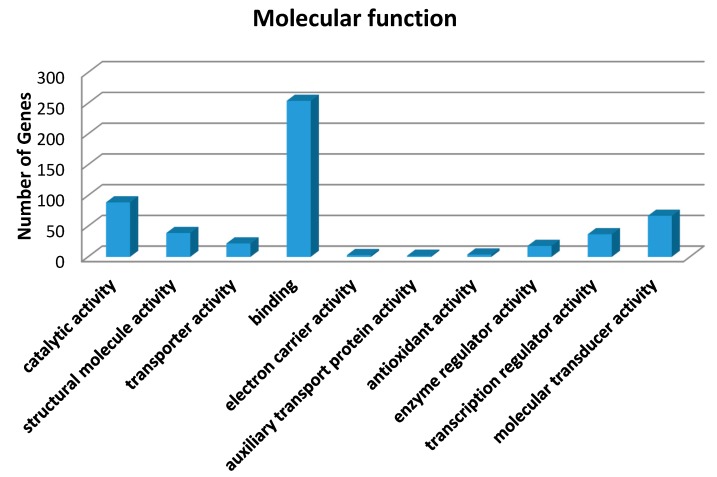

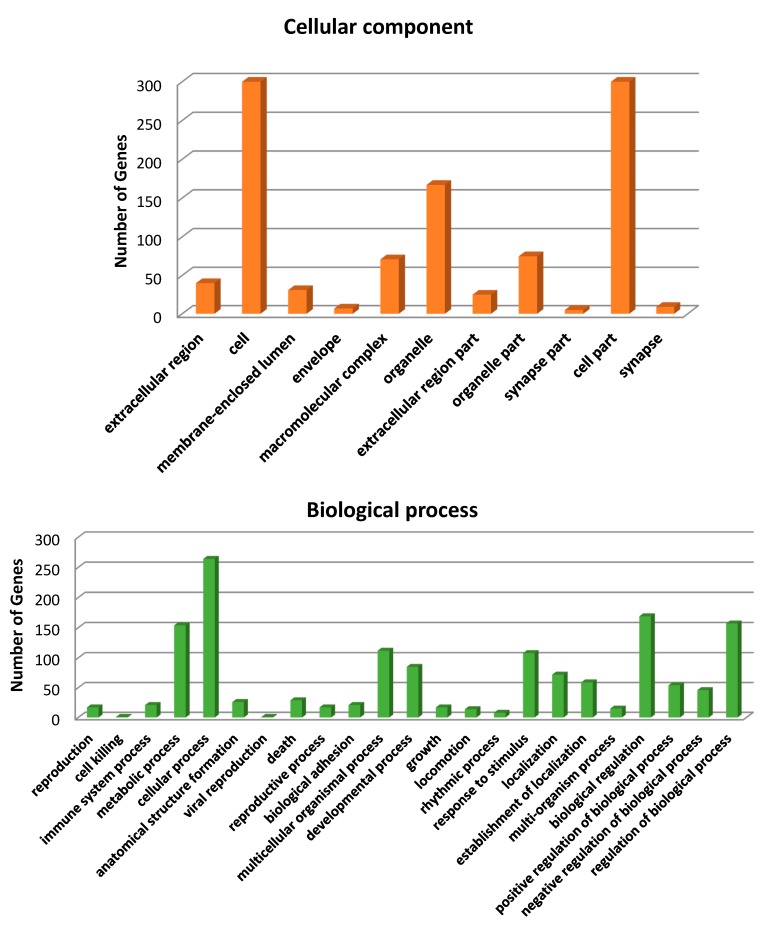
Gene ontology (GO) analysis of 973 genes in which SM-induced alteration of expression was abolished or attenuated by MSTF treatment. These genes of liver tissue were subject to GO analysis using the SBC (Shanghai Biotechnology Corporation) Analysis System (http://sas.ebioservice.com/portal/root/molnet_shbh/index.jsp) and the results summarized in a bar chart showing the number of genes associated with GO terms. GO (Level 2) for the transcriptome under molecular function, cellular component and biological process.

Gene ontology (GO) annotation was further performed for these genes in terms of biological process, molecular function and cellular component. The distribution of the genes in different GO categories at Level 2 is shown in Figure 4. The highly-represented GO terms were cellular process, biological regulation, metabolic process and response to stimulus for biological processes, binding, catalytic activity and molecular transducer activity for molecular function, cell parts, cells and organelles for the cellular component.

**Figure 5:**
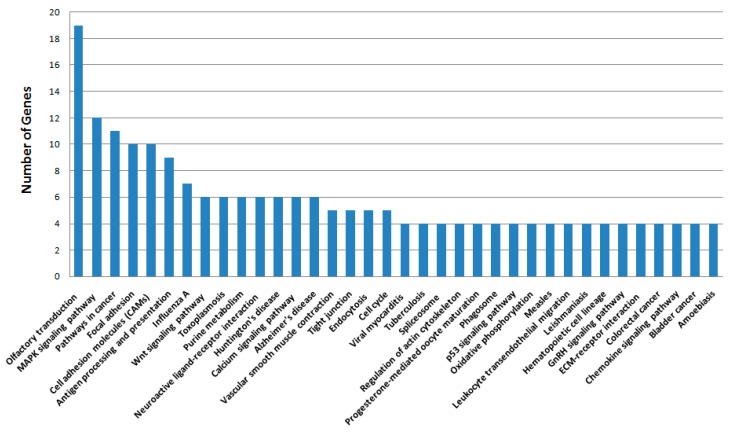
KEGG pathway analysis of 973 genes in which SM-induced alteration of expression was abolished or attenuated by MSTF treatment. These genes of liver tissue were subject to KEGG pathway analysis using the SBC Analysis System (http://sas.ebioservice.com/protal/root/molnet_shbh/index.jsp) and the results summarized in a bar chart showing the number of genes associated with each cellular pathway/process (*n* ≥ 4 genes).

Pathway enrichment analysis indicated that these genes were mainly involved in a total of 36 pathways, such as the MAPK signaling pathway, pathways in cancer, focal adhesion, cell adhesion molecules (CAMs), antigen processing and presentation, purine metabolism and the p53 signaling pathway (Figure 5). For example, the 12 genes presented in Table 2 were involved in MAPK pathways. Other pathways examined include: pathways in cancer (Table 3: 10 genes), focal adhesion (Table 4: 10 genes), cell adhesion molecules (CAMs) (Table 5: 10 genes) and antigen processing and presentation (Table 6: 9 genes).

**Table 2:** List of 12 genes involved in the MAPK signaling pathway. These SM-induced alterations of expression were abolished or attenuated by MSTF treatment.

Gene ID	Symbol	Description	Fold Change	
			SM	SM + MSTF
170579	*Fgf22*	fibroblast growth factor 22	1.74	1.21
54234	*Cacna1e*	calcium channel, voltage-dependent, R type, α 1E subunit	1.53	1.01
84489	*Fgfr3*	fibroblast growth factor receptor 3	2.35	0.9
292763	*Map4k1*	mitogen-activated protein kinase kinase kinase kinase 1	0.66	0.99
114495	*Map2k6*	mitogen-activated protein kinase kinase 6	1.52	0.76
94202	*Ptprr*	protein tyrosine phosphatase, receptor type, R	1.63	1.01
361679	*Dusp8*	dual specificity phosphatase 8	0.56	0.67
294254	*Hspa1b*	heat shock 70-kD protein 1B (mapped)	0.24	0.46
24516	*Jun*	jun proto-oncogene	0.64	0.67
24471	*Hspb1*	heat shock protein 1	1.72	1.36
24468	*Hspa8*	heat shock 70-kDa protein 8	0.47	1.03
171337	*Rap1b*	RAP1B, member of the RAS oncogene family	0.65	0.82

**Table 3:** List of 10 genes involved in pathways in cancer. These SM-induced alterations of expression were abolished or attenuated by MSTF treatment.

Gene ID	Symbol	Description	Fold Change	
			SM	SM + MSTF
691155	*Ccdc6*	coiled-coil domain containing 6	1.66	1.32
58919	*Ccnd1*	cyclin D1	0.56	0.73
24337	*Erbb2*	v-erb-b2 avian erythroblastic leukemia viral oncogene 2	0.6	0.69
25163	*Cdkn2a*	cyclin-dependent kinase inhibitor 2A	1.67	1.19
170579	*Fgf22*	fibroblast growth factor 22	1.74	1.21
84489	*Fgfr3*	fibroblast growth factor receptor 3	2.35	0.9
114517	*Itga6*	integrin, α 6	1.51	1.08
24516	*Jun*	jun proto-oncogene	0.64	0.67
25682	*Ppard*	peroxisome proliferator-activated receptor δ	1.57	0.88
363595	*Tcf7*	transcription factor 7 (T-cell specific, HMG-box)	1.68	1.02

**Table 4:** List of 10 genes involved in focal adhesion. These SM-induced alterations of expression were abolished or attenuated by MSTF treatment.

Gene ID	Symbol	Description	Fold Change	
			SM	SM + MSTF
29161	*Cav3*	caveolin 3	1.61	0.76
58919	*Ccnd1*	cyclin D1	0.57	0.73
29393	*Col1a1*	collagen, type I, α 1	0.51	0.49
84352	*Col1a2*	collagen, type I, α 2	0.65	0.76
114517	*Itga6*	integrin, α 6	1.51	1.08
24516	*Jun*	jun proto-oncogene	0.64	0.67
24337	*Erbb2*	v-erb-b2 avian erythroblastic leukemia viral oncogene 2	0.6	0.7
362973	*Parvb*	parvin, β	1.57	1.2
171337	*Rap1b*	RAP1B, member of RAS oncogene family	0.65	0.82
116669	*Vwf*	von Willebrand factor	0.5	0.78

**Table 5:** List of 10 genes involved in cell adhesion molecules. These SM-induced alterations of expression were abolished or attenuated by MSTF treatment.

Gene ID	Symbol	Description	Fold Change	
			SM	SM + MSTF
24931	*Cd8b*	CD8b molecule	1.53	1.02
65129	*Cldn1*	claudin 1	0.64	0.96
315953	*Cldn18*	claudin 18	1.94	0.99
287099	*Cldn9*	claudin 9	0.61	0.59
114517	*Itga6*	integrin, alpha 6	1.51	1.08
171297	*Nlgn3*	neuroligin 3	1.5	0.88
24699	*Ptprc*	protein tyrosine phosphatase, receptor type, C	0.66	0.98
294269	*RT1-Da*	RT1 class II, locus Da	0.59	0.97
294270	*RT1-Db1*	RT1 class II, locus Db1	0.65	0.92
363930	*Selplg*	selectin P ligand	1.66	1.05

**Table 6:** List of 9 genes involved in antigen processing and presentation. These SM-induced alterations of expression were abolished or attenuated by MSTF treatment.

Gene ID	Symbol	Description	Fold Change	
			SM	SM + MSTF
294254	*Hspa1b/Hsp70*	heat shock 70 kD protein lB (mapped)	0.24	0.46
24468	*Hspa8/HSP70*	heat shock 70 kD protein 8	0.47	1.03
25599	*Cd74/Ii/SLIP/CLIP*	Cd74 molecule, major histocompatibility complex, class II invariant chain	0.62	0.81
24931	*Cd8b/CD8*	CD8b molecule	1.53	1.02
500338	*Klrc3/KIR*	killer cell lectin-like receptor subfamily C, member 3	0.51	0.77
29684	*Klrc2/KIR*	killer cell lectin-like receptor subfamily C, member 2	0.53	0.79
25110	*Klrd1/KIR*	killer cell lectin-like receptor subfamily D, member 1	0.44	0.96
294269	*RTl-Da/MHCII*	RTl Class II, locus Da	0.59	0.97
294270	*RTl-Db1/MHCII*	RTl Class II, locus Db1	0.65	0.92

**Figure 6:**
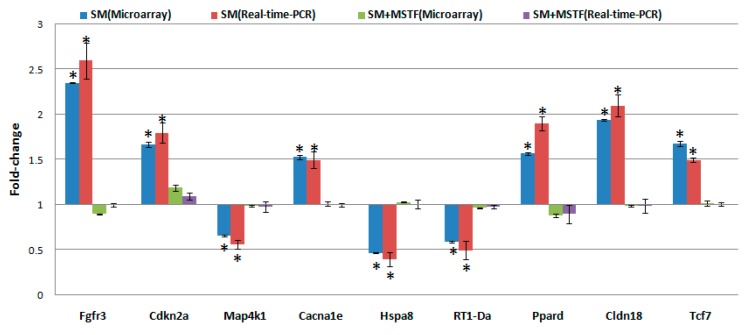
Quantitative real-time PCR confirmation of the microarray data. qRT-PCR was performed on nine genes that showed differential expression in treatment groups (SM, SM + MSTF) *versus* untreated controls. Gene expression levels are shown as the mean normalized to the expression of the housekeeping gene β-actin. Each sample was measured in triplicate. Columns, mean of three or four rats in the microarray experiment or a mean of five rats in PCR; bars, SD. Asterisks indicates statistical significance compared to the control (non-SM exposed rats) by the *t*-test (*p* < 0.05). The comparison of the fold change produced by the microarray with the relative expression ratio obtained from real-time PCR, with good concordance.

To validate the consistency of microarray analysis in the present study, we compared the gene expression levels of selected genes between microarray and real-time PCR. We determined the mean value of expression of the selected genes in five independent rats from each exposure group. This was compared to those in pooled RNA from five non-SM-exposed rats. The qualitative changes in gene expression levels were consistent between these analyses (Figure 6).

## 3. Discussion

Although oxidative stress and inflammation in vesicant-related injury are reported as major consequences of vesicant exposure, antioxidants, scavengers and anti-inflammatory drugs alone do not exhibit potent therapeutic effects [16,18]. This suggests that agents are needed with diverse activity towards multiple pathways, including targeting oxidative stress and inflammation. MSTF is the mixture of salvianolic acids (SA) of S*alvia miltiorrhiza* and total flavonoids (TFA) of *Anemarrhena asphodeloides*. SA is the most abundant water-soluble phenolic compound extracted from Radix S*alvia miltiorrhiza* (Danshen). Due to the polyphenolic structure, SA was thought to be comprised of free radical scavengers. Indeed, SA was found to have potent anti-oxidative capabilities *in vitro* and *in vivo* [19]. SA possessed a variety of biological and pharmaceutical activities, such as protecting effects against impaired vascular responsiveness in streptozotocin-induced diabetic rats, the cardioprotective effects on animal models of heart hypoxia/reoxygenation injury, the antiplatelet and antithrombotic activities in an arterio-venous shunt model and protective effects against carbon tetrachloride or concanavalin A-induced acute liver damage [20,21,22,23,24]. Total flavonoids (TFA) were extracted from the rhizome of *Anemarrhena asphodeloides* (Zhimu) used as an antipyretic, anti-inflammatory, anti-diabetic and antidepressant in traditional Chinese medicine [25]. TFA is composed of 16 flavonoid compounds, including xanthones, mangiferin, ismangiferin, neomangiferin and 1,4,5,6,-tetrahydroxyxanthone. TFA showed valuable bioactivities, such as anti-oxidation, anti-virus, anti-inflammation, anti-skin aging and damage, as well as other activities [26,27]. Findings in the present study demonstrated the potent therapeutic efficacy of MSTF in attenuating SM toxic responses. The combination of different types of agents above in MSTF can benefit from each other with different roles in the formula and ultimately gain the goal of enhancing efficacy, which is consistent with the core thinking of traditional Chinese medicine theory. To our knowledge, this is the first time that such a combination has been tested in therapeutic efficacy on SM toxicity.

The threat of warfare mustard vesicants’ exploitation also as potential terrorist agents poses challenges to vesicant research to develop effective medical countermeasures to vesicant injuries [18]. SM-induced injuries can take several months to heal and require long-term medical treatment. Exposure to SM triggers an array of complex signal transduction pathways, suggesting the need for pleiotropic agents or combination therapies to treat SM-induced injuries. The present study revealed that the treatment with MSTF significantly increased the survival levels of rats (Figure 1) and attenuated the SM-induced morphological changes in the testis, small intestine and liver tissues (Figure 2), confirming the potent therapeutic efficacy of MSTF in attenuating SM-induced toxic responses.

Exposure to SM or CEES (2-chloroethyl ethyl sulfide, SM analog) has been shown to deplete glutathione (GSH) stores *in vitro* and *in vivo* [13,28,29]. GSH depletion may inhibit further clearance of SM and, importantly, elicit oxidative stress, lipid peroxidation and macromolecular damage [10,28,30]. In line with these ideas, supplementation with GSH or GSH analogs protected against CEES- or SM-induced cytotoxicity in a number of cell lines [15], whereas blocking GSH synthesis rendered cells more sensitive to mustard toxicity [13]. Treatment with GSH was shown to increase the viability of keratinocytes exposed to CEES [7]. Consistent with these, in this study, MSTF treatment 1 h after SM subcutaneous injection significantly elevated the levels of GSH compared to the SM-only group, which strongly suggests that MSTF improves the animal survival level and inhibits the toxic effects of SM by elevating the levels of GSH. A previous study reported the dose- and time-dependent effects of SM on the antioxidant system and lipid peroxidation in liver and brain of rats [31]. At SM doses lower than 10 mg/kg after two and seven days of exposure, no significant change in GSH and malondialdehyde (MDA) levels were observed [31]. In this study, at a single dose of 3.5 mg/kg SM after seven days of exposure, no significant changes in superoxide dismutase (SOD) and MDA levels were observed.

The liver was considered to be a major target organ of SM-induced toxicity either after a single dose or repeated doses [17]. To gain insight into the molecular mechanism of the therapeutic effects of MSTF on SM toxicity, gene expression analysis was conducted on rats liver tissues using microarrays. In the present study, gene expression profiles revealed a dramatic effect of MSTF on alterations of gene expression caused by SM. Interestingly, MSTF treatment prevented SM-induced differential expression of 93.8% (973 genes) of genes. We focused on these genes regulated by SM, and these alterations of expression were abolished or attenuated by MSTF treatment, as these genes might be regulated by MSTF and involved in reversing the toxic effects of SM exposure. Gene ontology mapping of these gene sets revealed several major biological processes, which were cellular process, biological regulation, metabolic processes and response to stimulus (Figure 4). Pathway enrichment analysis indicated that these genes were mainly involved in a total of 36 pathways (Figure 5). Many of these pathways were reported to be induced by SM in the previous studies, such as the MAPK signaling pathway [32,33,34,35,36], pathways in cancer [32], antigen processing and presentation [37], cell adhesion molecules (CAMs) [36,37], cell cycle [32], the p53 signaling pathway [32,36], vascular smooth muscle contraction [32], progesterone-mediated oocyte maturation [32], hematopoietic cell lineage [32,36], neuroactive ligand-receptor interaction [36], purine metabolism [32,36], chemokine signaling pathway [32], tight junction [32], bladder cancer [32] and amebiasis [32]. Oxidative stress and inflammation in vesicant-related injury were reported as major consequences of SM exposure [16,18]. MAPK signaling pathways play critical roles in responding to oxidative stress and inflammation [38]. Interestingly, eight of nine SM-induced genes in antigen processing and presentation were significantly downregulated, and these downregulations were abolished or attenuated by treatment with MSTF (Figure 6). In addition, the previous study reported that silibinin (C_25_H_22_O_10_), a flavanone isolated from the seeds of *Silybum marianum,* attenuated sulfur mustard analog (CEES)-induced skin injury by targeting multiple pathways [16]. These data suggest that MSTF attenuates SM-induced injury by targeting multiple pathways, including MAPK signaling pathways, as well as antigen processing and presentation. These studies further give insight into the antioxidant and potential pleiotropic mechanisms of MSTF in reversing the toxic effects of SM. MSTF should be further assessed as a potential therapeutic against SM-induced injuries.

## 4. Materials and Methods

### 4.1. Chemicals

SM was prepared by the Chemical Biological Defence Section (Second Military Medical University, Shanghai, China) and was found to be above 99% pure by gas chromatographic analysis. Salvianolic acids (SA) were isolated from Radix *Salvia miltiorrhiza* (Danshen) and obtained from Shanghai Winherb Medical Technology Co. (Shanghai, China). Total flavonoids (TFA) of *Anemarrhena asphodeloides* (Zhimu) were obtained from Guangzhou LifeTech Pharmaceuticals Co. (Guangzhou, China). MSTF was the mixture of SA and TFA (the weight ratio of SA:TFA is 1:4). All other chemicals used were of analytical grade (Sinopharm Chemial Reagent Co., Shanghai, China).

### 4.2. Animals

SD (Sprague Dawley, SD) male rats (200 ± 20 g) were purchased from SLAC laboratory Animal Co., Ltd. (SLAC, Shanghai, China) and divided randomly into several groups. Animals were kept under standard laboratory conditions of temperature, pressure, humidity and a 12-h photoperiod. All animal procedures were performed according to the Guide for the Care and Use of Laboratory Animals recommended by the National Institutes of Health and approved by the Ethics Committee for Animal Experimentation of the Second Military Medical University.

### 4.3. Subcutaneous Injection with SM and Drug Administration

Rats were randomly assigned to one of the five following treatment groups (10 rats per group): normal control, SM and SM + MSTF low, medium and high dose (30, 60, 120 mg/kg body weight/day). For the subcutaneous injection, SM was dissolved in propylene glycol, and 0.1 mL were given using the gas-tight syringe on the back skin of the rats on a circular area of about 1.5 cm in diameter (the hair on the application site was closely clipped 24 h before SM application). The SM was applied as a single dose of 3.5 mg/kg body weight equivalent to 0.7 LD_50_ (7-day observation for mortality) [39,40]. The equivalent volume of solvent was given to control rats. MSTF (120 mg/kg) dissolved in double-distilled water was administered intragastrically 1 h after the application of SM, and after, the same dose of MSTF was given every 24 h. For the survival study, different doses of MSTF were administered to the rats once each day for 7 consecutive days 1 h after SM treatment. We monitored animals three times daily for a period of 7 days to determine survival rates, and moribund animals were euthanized according to humane endpoints. The clinical criteria of moribund are being in the state of dying with no expectation of recovery, where animals display a combination of the following: lowered body temperature, continuous shaking, hunched back, impaired or slow motion and inability to maintain sternal recumbency [41]. Moribund animals were placed in a separate cage with CO_2_ until no breathing was observed, followed by a cervical dislocation as a secondary confirmatory method of euthanasia. Any surviving animals at the end of the study were also subjected to euthanasia by the application of CO_2_ followed by cervical dislocation.

After 7 days, blood samples were obtained from periorbital sinus vein for the evaluation of oxidative markers, such as superoxide dismutase (SOD), reduced glutathione (GSH) and malondialdehyde (MDA).

### 4.4. Enzyme Activity Assay

Blood obtained from the periorbital sinus vein was collected and centrifuged at 3000 rpm for 10 min at 4 °C. The supernatant was used to perform the antioxidant enzyme assays using commercially available ELISA kits (Cayman Chemical Co., Ann Arbor, MI, USA). The plates were read at 450 nm with a correction wavelength of 550 nm on the BioTek Synergy 4 Multi-Mode Microplate Reader (BioTek Instruments, Inc., Winooski, VT, USA).

### 4.5. Sample Collection and Histopathologic Examination

Seventy-two hours following injection with SM, the rats were anesthetized and sacrificed by cervical dislocation. For testis, small intestine and liver histopathology analysis, the sections of tissue were processed for light microscopy. This processing consisted of specimen fixation in 4% buffered neutral formalin solution for 1–2 days, embedding in paraffin wax, slicing sections (5 μm) and staining them with hematoxylin and eosin (H&E). Histopathologic examination was blindly evaluated by a pathologist. In addition, 24 h following injection with SM, the rats were sacrificed. Liver tissues of all groups were processed for the microarray experiment.

### 4.6. Gene Expression Microarray and Data Analysis

The microarray experiments were performed as described previously [42]. The 4 × 44 K Whole Rat Genome Oligo Microarray (Agilent Technologies, Santa Clara, CA, USA) was hybridized with Cy3-labeled cRNA using the Gene Expression Hybridization Kit (Agilent Technologies) in a hybridization oven (Agilent Technologies), according to the manufacturer’s instructions. Raw data were obtained by Feature Extraction software 10.7 (Agilent Technologies) and normalized by the Quantile algorithm, Gene Spring Software 11.0 (Agilent Technologies). The microarray experiments were conducted at the National Engineering Center for Biochip in Shanghai, China, according to the procedures in the Agilent technical manual. After normalization, genes in the treatment groups with at least a 1.5-fold change in expression were considered as upregulated or downregulated in comparison to non-treated groups (control). To determine significant proportions of differentially-expressed genes within treated groups, the hypergeometric probability *p* was calculated. *p* < 0.05 was considered significant.

Microarray data analysis was performed using the SBC Analysis system, which is available on the website: http://sas.ebioservice.com/protal/root/molnet_shbh/index.jsp. The username and password to access this website are available upon request. A general description of the SBC analysis system can be found on the website: http://www.ebioservice.com/eng/index.asp. The microarray data generated in this study have been deposited in the Gene Expression Omnibus (GEO) database under Accession Number GSE62994.

### 4.7. Quantitative Real-Time-PCR Array Validation

qRT-PCR was performed essentially as described previously [42]. In total, nine genes were chosen for RT-PCR validation. PCR primers (Table 7) were designed to either span or flank introns by using the ProbeFinder Version 2.50 (http://www.roche-applied-science.com). Data are presented as the mean ± SD.

**Table 7:** Oligonucleotides primers used in this study for quantitative real-time-PCR analysis. The primer sequences were designed by using the ProbeFinder Version 2.50 (http://www.roche-applied-science.com).

Accession No.	Symbol	Primer	Sequence
NM_053429	Fgfr3	Forward	acgccctacgtcactgtactc
		Reverse	gaacctctagctccctgtcg
NM_031550	Cdkn2a	Forward	cagattcgaactgcgagga
		Reverse	tgcagtactaccagagtgtctagga
NM_001106243	Map4k1	Forward	cgcctgtctttcattggaac
		Reverse	cacctttcagggccacag
NM_019294	Cacna1e	Forward	taccgcgcctggatagac
		Reverse	gctgatgttcccgagttttt
NM_024351	Hspa8	Forward	gcacaggaaaggagaacaagat
		Reverse	catgcgctcaatatcctcct
NM_001008847	RT1-Da	Forward	aacgcaacgcagtggaac
		Reverse	tcaatgagctctcacggaag
NM_013141	Ppard	Forward	ggaccagagcacacccttc
		Reverse	gaggaaggggaggaattctg
NM_001014096	Cldn18	Forward	gtgccggccatacttcac
		Reverse	atgcccacgatcatcagg
XM_001073458	Tcf7	Forward	tcccccatactgtgagctg
		Reverse	ggcagggaagtgctgtctat
EF156276	β-actin	Forward	cccgcgagtacaaccttct
		Reverse	cgtcatccatggcgaact

### 4.8. Statistical Analysis

Data were presented as the mean ± SD. One-way analysis of variance (ANOVA) followed by Dunnett’s test was used to examine differences among untreated (control) and treated (SM, SM + MSTF) groups. Differences were considered significant when *p* < 0.05.

## 5. Conclusions

Treatment with MSTF after subcutaneous injection with 3.5 mg/kg SM significantly increased the survival levels of rats and attenuated the SM-induced morphological changes in the testis, small intestine and liver tissues. Treatment with MSTF at doses of 60 and 120 mg/kg caused a significant (*p* < 0.05) reversal in SM-induced GSH depletion. Gene expression profiles revealed that treatment with MSTF had a dramatic effect on gene expression changes caused by SM. Treatment with MSTF prevented SM-induced differential expression of 93.8% (973 genes) of 1037 genes. Pathway enrichment analysis indicated that these genes were mainly involved in a total of 36 pathways, such as the MAPK signaling pathway, pathways in cancer, antigen processing and presentation. These data suggest that MSTF attenuates SM-induced injury by increasing GSH and targeting multiple pathways, including the MAPK signaling pathway, as well as antigen processing and presentation. These results suggest that MSTF has the potential to be used as a potential therapeutic agent against SM injuries.

## Supplementary Materials

Supplementary materials can be found at http://www.mdpi.com/1422-0067/16/10/24555/s1.
